# Systematic Reviews in Neonatal Respiratory Care: Are Some Conclusions Misleading?

**DOI:** 10.3389/fped.2020.00007

**Published:** 2020-01-31

**Authors:** Andres Maturana, Fernando Moya, Steven M. Donn

**Affiliations:** ^1^Neonatology, Clinica Alemana, Department of Pediatrics, Santiago, Chile; ^2^Faculty of Medicine, Centro de Desarrollo Educacional, Universidad del Desarrollo, Santiago, Chile; ^3^Betty Cameron Children's Hospital, Coastal Carolina Neonatology, Coastal Children's Services, PLLC, Wilmington, NC, United States; ^4^Division of Neonatal-Perinatal Medicine, C.S. Mott Children's Hospital, Michigan Medicine, Ann Arbor, MI, United States

**Keywords:** neonatal respiratory care, meta-analysis, systematic reviews, clinical decision-making, infant-newborn

## Abstract

An increasing amount of information is currently available in neonatal respiratory care. Systematic reviews are an important tool for clinical decision-making. The challenge is to combine studies that address a specific clinical question and have similar characteristics in terms of populations, interventions, comparators, and outcomes, so that their combined results provide a more precise estimate of the effect that can be validly extrapolated into clinical practice. The concept of heterogeneity is reviewed, emphasizing that it should be considered in a wider perspective and not just as a mere statistical test. A case is made of how well-designed studies of the neonatal respiratory literature, when equivocally combined, can provide very precise but potentially biased results. Systematic reviews in this field and others should be rigorously peer-reviewed before publication to avoid misleading readers to potentially biased conclusions.

## Introduction

We are currently confronted with an overwhelming amount of information in all medical disciplines, and neonatal care is no exception (1, 2). A systematic review of the current literature can provide information that may be combined, thus increasing statistical power and providing a quantitative estimate of the effect in a meta-analysis (3). Although systematic reviews addressing a specific clinical question can help clinicians appraise in a summarized format all or most of the existing research pertaining to that topic and aid in bedside decision-making, they have recognized limitations (4, 5). Clinicians are sometimes confronted with systematic reviews that claim results based on combining studies that differ in substantial ways and therefore yield conclusions that are very difficult to interpret (6). Most of us would agree that almost any respiratory outcome in premature infants could be significantly influenced by antenatal steroid exposure and gestational age. Nevertheless, systematic reviews combining study populations with significant differences in these relevant variables have been published (**Table 2**).

The purpose of this review is to raise awareness of the importance of adequately appraising systematic reviews, using examples from the neonatal respiratory literature that, in our view, can sometimes lead to misleading conclusions. Table 1 summarizes the definitions of terms that will be used.

**Table 1:** Terms used in this review.

**Term**	Definition (^*^)
Systematic Review	The identification, selection, appraisal, and summary of primary studies that address a focused clinical question using methods to reduce the likelihood of bias.
Meta-Analysis	A statistical technique for quantitatively combining the results of multiple studies that measure the same outcome into a pooled or summary estimate.
Heterogeneity	Differences among individual studies included in a systematic review. These differences can refer to study characteristics or study results.
*I*^2^ Statistic	The *I*^2^ statistic is a test of heterogeneity. The results range from 0 to a 100% indicating no heterogeneity to high heterogeneity, respectively.
Bias	Systematic deviation from the truth because of a feature of the design or conduct of a research study. This can skew the outcome in a certain direction.
Selection Bias	Occurs when the population that is selected for a study is not representative of the general population addressed by the question the study intends to answer. This has the consequence that study results although not necessarily biased may not be applicable to the general population.

* *Definitions adapted from Users' Guides to the Medical Literature: Essentials of Evidence-Based Clinical Practice, Third Edition (7)*.

## The Concept of Heterogeneity

A systematic review summarizes the existing research that addresses a specific clinical question in a systematic and reproducible way. For the purpose of this review, we will refer to systematic reviews addressing the effect of therapeutic interventions in randomized clinical trials. In some cases, the studies found in the review process can be combined using meta-analysis, so as to provide a single more precise estimate of the effect (3). This entails some assumptions about the studies included in the analysis. First, the magnitude and direction of the treatment effect across the different studies should be relatively similar and that there are no significant variations in the results that could be explained by relevant differences among the studies. The studies should be combined only if they lack significant bias, if they answer the same specific question, if they include similar populations, and if they attempt to compare similar interventions and measure equivalent outcomes, so that a pooled effect of the results from individual studies yields a more precise and representative estimate of the treatment effect (6). The challenge is how much difference (heterogeneity) we are willing to tolerate in these parameters among the different studies without compromising the confidence of the pooled estimate. The usual approach to this conundrum is to evaluate heterogeneity in a statistical manner. Any of the tests used for this purpose are only providing information about differences between study results and telling us how likely the differences in individual trial results are from chance alone (9). A frequently used test for evaluating heterogeneity is the *I*^2^ statistic that estimates the heterogeneity as the magnitude of variability. It is easily interpreted as the percentage of heterogeneity in the point estimates from individual studies. When it approaches 0%, the reader can be relatively confident that any differences between the individual point estimates of the included studies is explained merely by chance and, therefore, the summary estimate of the treatment effect is credible. When this percentage approaches 100% the probability that only chance explains these differences is substantially less likely and, therefore, a summary effect is more difficult to interpret (10). The problem is that sometimes we can be confronted with differences in study design that make any pooled estimate of the effect difficult to interpret or even meaningless, and are not necessarily detected by any statistical test for heterogeneity. Therefore, heterogeneity between studies in a meta-analysis needs to be examined as much more than a simple statistical test, and clearly, one more relevant issue when critically appraising a systematic review.

## Heterogeneity in Included Populations, Interventions, Control Groups, and Outcomes

If we are considering therapeutic interventions, a certain homogeneity in the populations included in the different studies considered in a systematic review can be a very relevant issue. We should not feel comfortable drawing any conclusions from a meta-analysis within a systematic review that combines studies including populations that differ in characteristics that could potentially influence the magnitude or direction in the effect of the intervention being studied.

A systematic review by Ferguson et al. addressing the question of interventions to improve rates of successful extubation in preterm infants can help exemplify this point (8). If we review the comparison between high flow nasal cannula and nasal continuous positive airway pressure (CPAP) on the outcome respiratory failure, three studies are included in this analysis (Table 2) (11–13). As an example, the populations in the study by Yoder include more mature infants (>28 weeks) and with a significantly lower percentage of antenatal steroid receipt (<35%) than the other two included studies, and these are two well-recognized prognostic factors for respiratory failure. Fortunately, in this case we are alerted by an *I*^2^ of 55%, suggesting that chance does not adequately explain the variability between the point estimates. Regretfully, this is not always the case.

**Table 2:** Heterogeneity in populations included in the Meta-Analysis by Ferguson et al. (8).

**Trial**	**Mean gestational Age** **(treated/controls)**	**% of Antenatal Steroids** **(treated/controls)**	**% of Caffeine use** **(treated/controls)**
Yoder et al. (11)	33.5 ± 3.6/33.2 ± 3.2	38/32	27.0/30.0
Manley et al. (12)	27.7 ± 2.1/27.5 ± 1.9	93.4/94.7	99.3/98.0
Collins et al. (13)	27.9 ± 1.9/27.6 ± 1.9	88.0/89.0	100.0/100.0

An intervention will have an effect that will reflect a magnitude and a direction. Evidently, this is dependent upon the comparative intervention. It would not be correct to claim a certain magnitude of effect of a certain intervention if it is being compared to anything different than the standard of care for the control group, since this could potentially overestimate the real effect of the intervention. It would not make much sense to combine studies that have different comparators in a meta-analysis. A recently published systematic review by Wu et al. addresses the outcomes of surfactant administration in a minimally invasive way (via thin endotracheal catheter) to spontaneously breathing infants (14). In this review, four studies are included for the outcome of requiring mechanical ventilation within the first 72 h of life (15–18). The trial by Göpel compared a less invasively administered surfactant (LISA) to intubation and rescue surfactant via endotracheal tube in the control group, while Kanmas and Bao compared LISA with the Intubation-Surfactant-Extubate (INSURE) procedure in the control group. In these studies, specific criteria for respiratory failure where defined in the protocols. The included study by Kribs used LISA and compared this to surfactant administration with mechanical ventilation. In this last case, indications for mechanical ventilation were defined by protocol for the control group and, in fact, only one infant was not mechanically ventilated. For this analysis, the *I*^2^ statistic shows 0% heterogeneity, suggesting that the summary point estimate is not biased by any relevant differences between the studies. Nevertheless, it is obvious that these studies are completely different and probably should not have been combined for this outcome.

When evaluating the impact of an intervention on a specific outcome across different studies, an important assumption is that the outcome in each of the studies was similarly defined, so as to render the combined effect in a meta-analysis interpretable. This is particularly relevant when considering physician-driven outcomes, which are those that depend upon the treating physician and therefore rely on how every protocol in each study defined the criteria for this outcome. An example of such an outcome in neonatal practice is nasal CPAP failure or intubation for mechanical ventilation. We can expect differences in clinical practice among different centers and even within a single center among different clinicians. When one performs a systematic review, one forgoes the ability to conduct logistic regression analysis using center effect as a variable. The problem arises when we try to interpret combined results of studies that have, for instance, significant differences in the criteria for intubation, especially if it is not defined *a priori* in the various studies included in the systematic review.

Another example of this is the recently published review by Conte that addresses the comparison of high flow nasal cannula and nasal CPAP as the initial strategy to treat RDS in preterm infants (19). In this review, six studies are included in the analysis for the outcome of respiratory failure, but only five of them contribute with outcomes (11, 20–23). If we look at the *I*^2^ statistic, it shows that there is relatively little heterogeneity (17%) within the included studies for this outcome and, therefore, we should be fairly confident in interpreting this summary estimate of the treatment effect. Unfortunately, this statistic can only detect the mathematical heterogeneity in the individual point estimates of the effect but will not reflect relevant differences within the studies. In this example, three of the studies (20, 22, 23) have intubation thresholds utilizing an FiO_2_ of 0.4, whereas Nair and Karna (21) and Yoder et al. (11) have significantly higher thresholds for intubation (0.6 and 0.7, respectively). These differences will evidently bias the results toward a lower difference between the groups for this outcome, since fewer patients will meet the threshold. If we exclude these two studies, the analysis yields a significantly greater magnitude in the point estimate against using high flow nasal cannula as the initial support strategy (1.72 vs. 1.57).

## Limitations in Generalizability

When examining the conclusions of any trial, including those conducted under high standards, they can only provide an answer to a clinical question that generally is fairly specific (primary outcome), and applicable to the population studied. Good examples of this paradigm are those studies that compared CPAP at or soon after birth vs. intubation with or without surfactant administration. For instance, the COIN trial enrolled preterm infants of a minimum gestational age of 25 weeks or more, who were spontaneously breathing at 5 min of life (24). Therefore, their findings *do not* apply to all infants born at 25 weeks or more, but obviously more to those who were in apparently better status immediately after delivery. Furthermore, their findings *do not* apply at all to preterm infants below 25 weeks. In fact, in the systematic review of Schmolzer et al. comparing CPAP to intubation (usually plus surfactant), only one trial enrolled infants <25 weeks' gestation (SUPPORT) (25, 26). In this large trial, essentially all extremely preterm infants for whom informed consent had been obtained antenatally were enrolled. This is an important difference compared to the other trials included in this systematic review, where a more select population of preterm infants was enrolled. The critical nature of this potential source of bias is clearly demonstrated by Rich et al. who reported outcomes of all infants that were eligible for the SUPPORT trial but *were not enrolled* (27). Undoubtedly, essentially all meaningful outcomes were worse among those infants, signaling a clear selection bias, albeit smaller than in other trials of this systematic review.

## A Plausible Explanation for Statistical Associations

When interpreting the pooled results of a systematic review, we should not accept the results without considering some logical explanation behind them. An example of this point can be made in relation to a recently published systematic review by King and colleagues (28). In this review, two interfaces to deliver nasal CPAP were compared and a total of seven studies met the inclusion criteria; however, only six of them were considered for the outcomes of nasal CPAP failure and bronchopulmonary dysplasia (BPD) (29–33). When we look at the pooled results for nasal CPAP failure within 72 h after initiation, we see a marginally significant result in favor of nasal mask vs. binasal prongs (Risk ratio 0.72, 95%. CI 0.53–0.97) without considerable heterogeneity (*I*^2^ of 16%). What is more promising is the fact that there is a significant difference again in favor of the nasal mask interface with a reduction in moderate to severe BPD, this time with moderate heterogeneity (*I*^2^ of 30%). Nevertheless, if we try to find a plausible explanation for this difference based on better effectiveness and less failure with the nasal mask, the results do not support this. The study by Say et al. is the major contributor to the difference observed in moderate to severe BPD, but it shows no difference in the failure rate between the compared nasal CPAP interfaces (33). This strongly suggests that this observed association probably occurred by chance and is not related to the intervention.

## Conclusions

Systematic reviews are in great demand and remain a significant contribution for clinical decision-making and effectively provide an updated and informative perspective of the current state of the literature in a specific topic but their results should be interpreted with care. The Cochrane Library, which in many ways has set the standards for systematic reviews in therapeutic interventions, has not been always able to keep the published reviews updated with sufficient promptness, thus creating a valid space for alternate versions of already published topics.

We have shown how well-designed studies can be equivocally combined in a meta-analysis and lead to biased summary point estimates of the effect. Heterogeneity among studies is a potential source of bias and may not always be detected by statistical tests. The latter aim to detect variability between study results but cannot detect relevant differences in design that could result in a meaningless conclusion from the combination of very different studies. This problem should be better described in the existing literature. Publication requirements for systematic reviews should be strengthened, following currently existing guidelines and undergo a rigorous peer-review process that considers some of the issues discussed previously. Clinicians should definitely be more aware of potential sources of bias when reading published systematic reviews to avoid being misled by only interpreting their conclusions.

## Author Contributions

AM: substantial contributions to the conception, analysis, and interpretation of the work. Drafting and revising the manuscript critically for important intellectual content. Agrees to be accountable for all aspects for all aspects of the work in ensuring that questions related to the accuracy of any part are appropriately resolved. FM and SD: substantial contribution to the analysis and interpretation of the work. Drafting and revising the manuscript critically for important intellectual content.

### Conflict of Interest

The authors declare that the research was conducted in the absence of any commercial or financial relationships that could be construed as a potential conflict of interest.
